# Emergency department flow in an optimized setting

**DOI:** 10.1186/1757-7241-21-S2-A3

**Published:** 2013-09-09

**Authors:** Lars L Stubbe Teglbjærg

**Affiliations:** 1 Svendborg Emergency Department, FAM Svendborg, OUH Svendborg Hospital, DK 5700 Svendborg, Denmark

## Background

The patterns of patient admission and discharge rarely reflects patient needs. The main reason is the way we manage processes such as ward rounds, operations, radiology, outpatient handling, inpatient tests etc. This results in variable length of stay (LoS) in the emergency departments, even among patients admitted with similar conditions. We have implemented structured time-driven patient handling with five key elements: 1. Compilation of acute patients in a single joint acute ward. 2. Fast Track treatment of minor injuries. 3. Within a four-hour time limit a specialist-level treatment-directing diagnosis and treatment plan has to be made for patients admitted for inpatient treatment. 4. A discharge plan has to be made for in-patients with an estimated date of discharge within the same 4-hour time limit. 5. Adjustment of staffing, operation capacity, laboratory and radiology service according to patient flow. The purpose of the change was to secure uniform, fast, high quality diagnosing, care and treatment for all acute patients, 24 hours a day.

The objective of this study was to describe effects of a structured, time driven approach on patient flow.

## Methods

Data were obtained from our patient administrative system (FPAS) and our patient logistic system (Cetrea Emergency). Main outcome measures were average time to treatment initiation in the Emergency Room (ER), percentage of patients who finishes treatment in the emergency department (ED) and LoS for patients in the ED.

## Results

Following actual intervention, registered average time to treatment initiation for ER patients decreased more than 50% to less than 30 minutes.

The percentage of patients registered as having been discharged directly from the emergency department was 79,3%.

Average LoS in the ED was 6,1 hours, 12,3 hours for inpatients and 2,8 hours for ER outpatients.

## Conclusion

Structured intervention reduced registered time to treatment initiation in this setting. The setting enables diagnosis and definitive treatment of most acute patients. The results suggest further work in the analysis of quantitative effects of structured process changes in the handling of acute patients. Whether structured flow with mandatory structured treatment and discharge plans enhances treatment quality or initiates a 'self-fulfilling prophecy' warrants further investigation.

